# A systematic review and meta-analysis of efruxifermin’s efficacy in improving liver fibrosis in patients with NASH/MASH

**DOI:** 10.3389/fphar.2025.1594091

**Published:** 2025-05-30

**Authors:** Shuzhai Li, Wangyuan Zou, Yingying Zhou, Wenjie Li, Wei Liao, Tao Li, Zhiming Zhang

**Affiliations:** ^1^ Department of Anesthesiology, The First People’s Hospital, The Affiliated Chenzhou Hospital, Hengyang Medical School, University of South China, Chenzhou, Hunan, China; ^2^ Department of Anesthesiology, Xiangya Hospital, Central South University, Changsha, Hunan, China; ^3^ Department of Critical Care Medicine, The First People’s Hospital, The Affiliated Chenzhou Hospital, Hengyang Medical School, University of South China, Chenzhou, Hunan, China

**Keywords:** efruxifermin, FGF21, MASH, non-alcoholic, steatohepatitis, fibrosis

## Abstract

**Background & Aims:**

Efruxifermin is a potential treatment for non-alcoholic steatohepatitis (NASH, now termed metabolic dysfunction-associated steatohepatitis, MASH). This study aimed to analyze the effectiveness of efruxifermin in improving liver fibrosis in patients with MASH.

**Methods:**

Systematic searches of PubMed, Cochrane Library, and Embase databases were conducted. Randomized controlled trials evaluating the efficacy of efruxifermin compared with placebo in patients with MASH were included. The primary outcome was the proportion of patients with improvement in liver fibrosis by 1 or more stages without worsening of MASH. The secondary outcomes were non-invasive biomarkers of fibrosis and treatment-emergent adverse events (TEAEs).

**Results:**

This meta-analysis included 4 studies with a total of 325 patients with biopsy-proven MASH and stage F1–F4 fibrosis. All studies reported histological outcomes. Compared to placebo, efruxifermin demonstrated a higher relative risk (RR) of 1.97 (95% CI 1.21 to 3.19, *I* ^*2*^ = 0%, *P* = 0.006) for achieving improvement in fibrosis by ≥ 1 stage without worsening of MASH. Furthermore, efruxifermin improved fibrosis-related non-invasive biomarkers (enhanced liver fibrosis [ELF]score, N-terminal type-III collagen pro-peptide [ProC3], and liver stiffness by FibroScan). However, efruxifermin was associated with an increased risk of adverse events compared to placebo, but this finding was not robust in sensitivity analysis.

**Conclusion:**

Efruxifermin may be a potential therapeutic for MASH-related fibrosis, with the available data indicating seemingly favorable tolerability.

**Systematic Review Registration:**

https://www.crd.york.ac.uk/PROSPERO/view/CRD42023491895, CRD42023491895

## 1 Introduction

Metabolic dysfunction-associated steatohepatitis (MASH), the most severe and progressive form of metabolic dysfunction-associated steatotic liver disease (MASLD), is defined by the presence of 5% or greater hepatic steatosis accompanied by inflammation and MASLD hepatocyte injury (ballooning), with or without fibrosis (Matteoni et al., 1999; Chalasani et al., 2018). MASH can progress to liver fibrosis and cirrhosis, and eventually lead to hepatocellular carcinoma. It has been the fastest-growing cause of hepatocellular carcinoma (HCC) in the United States, the United Kingdom, India, Germany, France, and the Middle East, and the prevalence of MASLD-related HCC is likely to increase with the prevalence of obesity and diabetes (Huang et al., 2021). In addition, the incidences of cardiovascular disease, chronic kidney disease, and extrahepatic malignancy were also significantly increased in MASH patients (Loomba et al., 2021). The global prevalence of MASLD is currently estimated to be between 25% and 30%, with 3%–5% of cases progressing to MASH accompanied by advanced fibrosis or cirrhosis (Younossi et al., 2016; Murag et al., 2021; Le et al., 2022). MASH may soon become a major indication for liver transplantation (Loomba et al., 2020). Lifestyle modifications, including diet and exercise, can improve liver histology in patients with MASLD (Wong et al., 2013; Vilar-Gomez et al., 2015). However, studies have shown that lifestyle changes are difficult to achieve and maintain due to a combination of genetic, epigenetic, behavioral, physical, and cultural issues (Bellentani et al., 2008; Centis et al., 2010; Stewart et al., 2015; Lean et al., 2018). Thus, to alleviate the burden of disease on individuals and society, it is imperative to identify suitable pharmaceutical interventions targeting MASH and its associated fibrosis (Konerman et al., 2018). Currently, the European and American Association for the Study of the Liver recommend vitamin E and the proliferator-activated receptor γ (PPAR-y) ligand pioglitazone only for selected patients (Peng et al., 2020). Resmetirom (Rezdiffra™), an oral thyroid hormone receptor-β agonist, received accelerated FDA approval in March 2024 for noncirrhotic metabolic dysfunction-associated steatohepatitis (MASH) with moderate-to-advanced liver fibrosis (F2–F3), demonstrating significant histological improvements in fibrosis regression and MASH resolution in phase 3 trials alongside manageable safety outcomes, though with a high incidence of gastrointestinal adverse events (e.g., diarrhea [27%–33%] and nausea [19%–22%] vs. 16% and 13% in placebo groups) (Souza et al., 2025). Currently, therapeutic options for MASH remain relatively limited, posing a significant challenge to the treatment of MASH. The therapeutic mechanisms of most current agents target the metabolic dysfunction of hepatocytes or inhibit inflammation and fibrosis (Brunt et al., 2019). However, one study indicated that an ideal therapeutic approach to treat advanced fibrosis and address the profibrotic environment driven by hepatocyte death (apoptosis) associated with chronic steatosis, lipotoxicity, and oxidative and endoplasmic reticulum stress (Harrison et al., 2021).

Fibroblast growth factors (FGFs) are a family of 22 signaling proteins that regulate reproduction, development, repairment, and metabolism (Beenken and Mohammadi, 2009; Maddaluno et al., 2017; Kliewer and Mangelsdorf, 2019). Fibroblast Growth Factor 21 (FGF21) is an endocrine member of the fibroblast growth factor FGF15/19 subfamily (Tillman and Rolph, 2020). It activates the cell membrane co-receptor complex of β-klotho and one of its homologous FGF receptors (FGFR), including FGFR1c, FGFR2c, or FGFR3c (Kurosu et al., 2007; Ogawa et al., 2007). This activation occurs through various pathways, such as mitogen-activated protein kinase (MAPK) and AKT signaling networks, facilitating downstream FGFR signal transduction (Ornitz and Itoh, 2015). In 2005, Kharitonenkov and colleagues discovered that FGF21 can induce glucose uptake in adipocytes, leading to a reduction in blood glucose levels (Kharitonenkov et al., 2005). Currently, analogs of FGF21 have emerged as promising therapeutic targets, possessing many characteristics ideal for the treatment of MASH (Harrison et al., 2023b). FGF21 typically acts directly or indirectly on multiple major organs, especially adipose tissue, the liver, and the brain (Tian et al., 2023). It can, to a certain extent, protect individuals from the effects of obesity, insulin resistance, metabolic abnormalities, and irregular vascular homeostasis (Kharitonenkov et al., 2005). FGF21 can also directly reduce lipid deposition in liver cells in a non-insulin-dependent manner, thereby hindering the development of MASLD (Tian et al., 2023). In both *in vitro* and *in vivo* liver fibrosis and MASH models, FGF21 receptor agonists inhibit liver inflammation, fat content, and hepatic fibrosis (Tian et al., 2023).

Efruxifermin, previously known as AKR-001 and AMG 876 (Kaufman et al., 2020; Shao and Jin, 2022), has a molecular weight of 92 kDa. It is a fusion protein consisting of the human IgG1 Fc domain and a modified human FGF21 (Fc-FGF21). This fusion protein exhibits balanced *in vitro* agonist potency for FGFR1c, FGFR2c, and FGFR3c14 (Stanislaus et al., 2017). Mutations in the specific FGF21 portion of efruxifermin result in a longer duration of action compared to most FGF21 analogs, with an extended half-life of 3–3.5 days and increased affinity for binding to the specialized co-receptor β-klotho (Stanislaus et al., 2017; Kaufman et al., 2020). Compared to pegbelfermin, efruxifermin demonstrates lower susceptibility to cleavage by fibroblast activation protein (FAP) (Tillman and Rolph, 2020), thus exhibiting improved *in vivo* stability and prolonged half-life. To date, efruxifermin has demonstrated significant advantages over other FGF21 analogs in enhancing insulin sensitivity, improving blood glucose control, and reducing hepatic fat (Tillman and Rolph, 2020).

A recently published phase 2b clinical trial indicated that efruxifermin appeared to be a promising therapeutic approach for treating patients with fibrosis due to MASH (Harrison et al., 2023a). However, a comprehensive systematic analysis of efruxifermin is yet to be reported. Therefore, we conducted this systematic review and meta-analysis to elucidate the effectiveness of efruxifermin in improving liver fibrosis in MASH patients. We believe that our research findings contribute to a deeper understanding of the value and potential of efruxifermin in practical clinical applications.

## 2 Material and methods

The study protocol was registered with the Prospective Register of Systematic Reviews (PROSPERO). This systematic review was conducted according to the Preferred Reporting Items for Systematic Reviews and Meta-Analyses (PRISMA) 2020 and AMSTAR (Assessing the methodological quality of systematic reviews) Guidelines (Shea et al., 2017; Page et al., 2021). This study differed from the protocol in two aspects: (1) using relative risk instead of odds ratio for binary variables pooling, as all included studies were randomized controlled trials (RCTs); (2) not analyzing NIS4 (a blood-based non-invasive test to determine the risk of MASH and NAS ≥4 and F ≥ 2 among patients with metabolic risk factors) due to insufficient data.

### 2.1 Eligibility criteria

**FIGURE 1:**
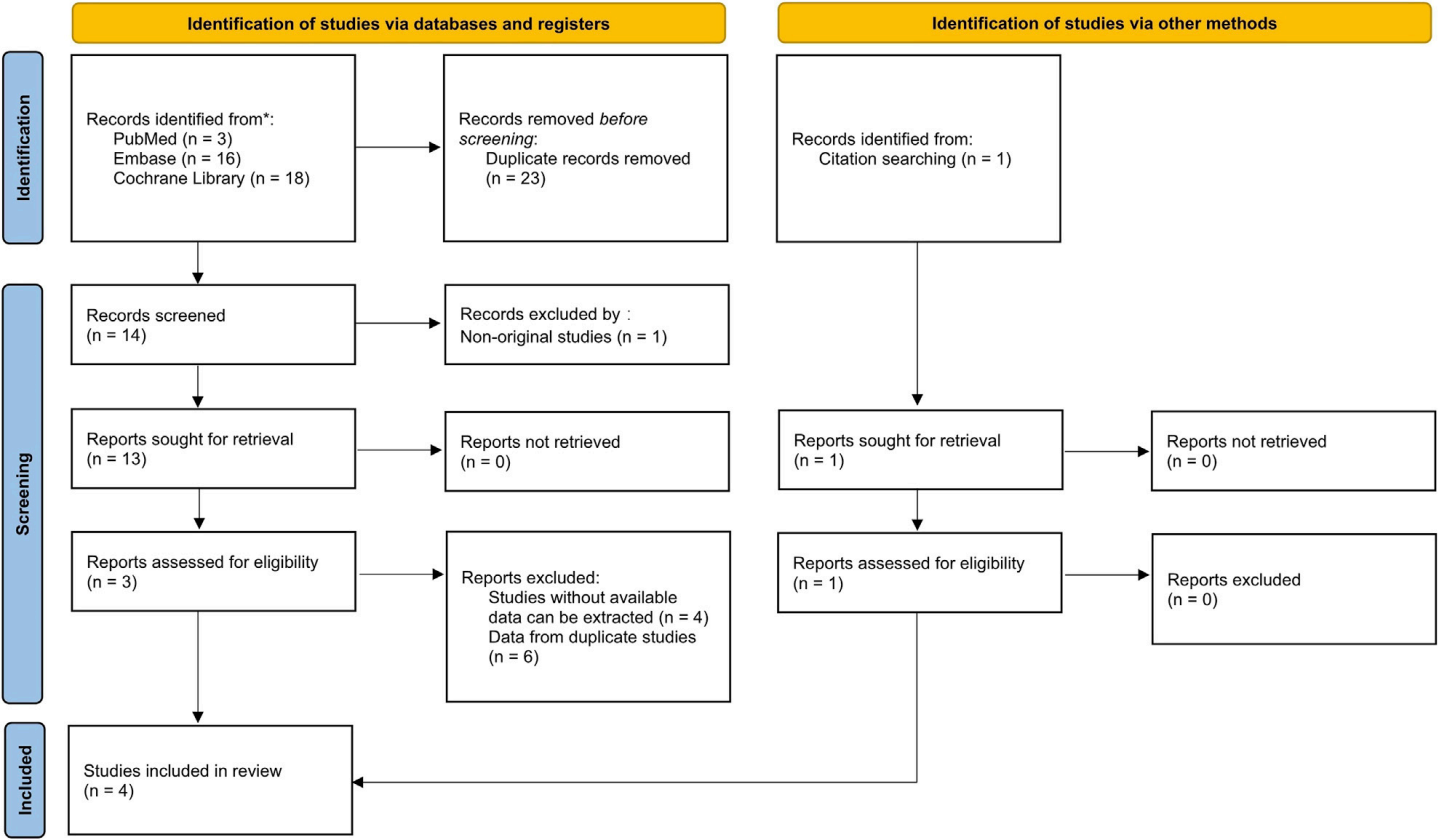
Flow diagram of study selection for the systematic review and meta-analysis. Initial database search (PubMed=3, Embase=16, Cochrane=18) identified 37 records. After removing 23 duplicates, 14 were screened. After excluding 1 non-original study, the full texts of 13 articles were reviewed, following which 10 were excluded (4 inaccessible data; 6 duplicates). One study added via citation searching, resulting in 4 studies included.

The inclusion criteria for studies were as follows: (1) the study design was an RCT; (2) studies in which patients were diagnosed with MASH using biopsy; (3) the intervention administered was efruxifermin; (4) the comparator was either placebo or another active therapy. The exclusion criteria were as follows: (1) duplicate literature; (2) non-human studies; (3) non-original studies (letters, reviews, editorials); (4) studies that did not include efruxifermin; (5) studies that did not include non-alcoholic steatohepatitis; (6) studies without available data can be extracted (Figure 1).

### 2.2 Data collection and retrieval strategies

We systematically conducted electronic searches for RCTs, irrespective of their publication status or year of publication. The most recent search was performed on 9 December 2023. Relevant studies were retrieved using the following databases: PubMed, Embase, and the Cochrane Library. Medical Subject Headings (MeSH) or the keywords “Non-alcoholic Fatty Liver Disease”, “efruxifermin” and “randomized” were used to search the literature without language restrictions. The specific search strategies are shown in Supplementary Tables S2–S4.

### 2.3 Outcome assessment

The primary outcome was the proportion of patients with improvement in liver fibrosis by 1 or more stages without worsening of MASH, defined as no increase in score for any one of the components of NAS—namely, ballooning, inflammation, or steatosis (Harrison et al., 2023a). Secondary outcomes included enhanced liver fibrosis (ELF)score, N-terminal type-III collagen pro-peptide (ProC3), and liver stiffness by FibroScan. Also included in the secondary data were treatment-emergent adverse events (TEAEs) and drug-related TEAEs, such as diarrhea, nausea, vomiting, and injection site reactions.

### 2.4 Selection process and data extraction

With strict adherence to previously established inclusion and exclusion criteria, two reviewers independently assessed all retrieved literature. The initial screening involved reviewing the titles and abstracts of all retrieved studies. Following the elimination of duplicates and studies not meeting the inclusion criteria, the remaining studies underwent further review, with the identification of potentially suitable studies achieved through a thorough examination of the complete texts.

Using a pre-designed table, two authors independently conducted data extraction. Demographic and outcome data were also collected. Specifically, we extracted the first author’s name and publication year, primary and secondary outcomes, clinical research phase, dosage of experimental drug, study duration, and basic characteristics of patients (number of patients, mean age, gender, race or ethnicity, metabolic risk factors and parameters, liver histology, markers of fibrosis, etc.) from each included study. Any disagreements were resolved by a senior researcher.

### 2.5 Data synthesis and statistical analysis

We used Review Manager (RevMan) 5.3 version (Cochrane Collaboration, Oxford, United Kingdom) to process the data for meta-analysis. For dichotomous variables, relative risk (RR) was used to express the effect size, while for continuous variables, mean difference (MD) or standardized mean difference (SMD) was used. If the standard deviation (SD) was not provided, standard error, 95% confidence interval (CI), or other relevant data were converted to SD accordingly. The following formula was used to convert standard error (SE) to SD: SD = SE × SQRT(n). Additionally, when only the 95% CI was available, SD was calculated using the following formula: SD = SQRT(n) × (Upper limit–Lower limit)/(TINV(1–0.95, n−1) × 2). When all outcome measurements across studies were based on the same scale, the mean difference (MD) was employed as the summary statistic. Otherwise, the standardized mean difference was used as the summary statistic. The heterogeneity of the included studies was analyzed using the Q statistic for the χ2 test (test level: α = 0.1), along with *I* ^*2*^ values to assess the level of heterogeneity. *I* ^*2*^ values <40%, 40%–60%, and >60% were respectively indicative of low, moderate, and significant heterogeneity. If statistical heterogeneity was present (*P* < 0.1 or *I* ^*2*^ ≥ 40%), the random-effects model was used for pooled effect size analysis. Otherwise (*P* ≥ 0.1 and *I* ^*2*^ < 40%), the fixed-effects model was employed for a more conservative estimation of the differences. Subgroup analyses were not performed due to the small number of included studies. Sensitivity analysis was performed using RevMan version 5.3. Publication bias was assessed using the funnel plot. We utilized the Grading of Recommendations Assessment, Development, and Evaluation (GRADE) system to evaluate the certainty of the outcomes.

### 2.6 Risk of bias in individual studies

The risk of bias in determining the quality of the included studies was assessed using the Cochrane Handbook for Systematic Reviews of Interventions. Two reviewers independently analyzed each study, evaluating the risk of bias across five domains: selection bias, performance bias, detection bias, attrition bias, and reporting bias. Each domain was categorized as low, high, or unclear risk. If the reported procedures met the criteria for low or high risk of bias within a domain, the study was classified accordingly. Unclear risk of bias was assigned when information was insufficient or uncertainty existed regarding bias likelihood (Higgins et al., 2011).

## 3 Results

### 3.1 Screening and characterization of studies

We identified a total of 37 studies from PubMed, Embase, and the Cochrane Library, of which 23 were duplicates. Among the remaining 14 studies, one was excluded due to being a non-original study, four were excluded due to lack of available data, and six were excluded due to data duplication. Additionally, one study was included based on relevant literature reading, resulting in the final inclusion of four studies (Harrison et al., 2021; Harrison et al., 2023a; Harrison et al., 2023b). Figure 1 illustrates the outcomes obtained from the literature screening procedure. The principal attributes of the studies selected for inclusion are summarized in Table 1.

**TABLE 1:** Characteristics of the included studies.

First author and year												
	Akero (2023)			Harrison et al. (2021)				Harrison et al. (2023a)		Harrison et al. (2023b)		
		Efruxifermin			Efruxifermin				Efruxifermin		Efruxifermin	
	Placebo (n = 61)	28 mg (n = 57)	50 mg (n = 63)	Placebo (n = 21)	28 mg (n = 19)	50 mg (n = 20)	70 mg (n = 20)	Placebo (n = 10)	50 mg (n = 20)	Placebo (n = 43)	28 mg (n = 42)	50 mg (n = 43)
Clinical research phase	2b			2a				2a		2b		
Study duration, week	36			12				16		24		
Mean age, years (SD)	61	62	59	52.4 (9.6)	50.4 (12.4)	52.6 (14.2)	53.0 (13.2)	57.1 (14.4)	61.1 (10.0)	55.0 (10.1)	56.5 (9.3)	52.4 (11.4)
Female, n (%)	38 (62)	39 (68)	44 (70)	15 (71)	10 (53)	10 (50)	11 (55)	3 (30)	16 (80)	27 (63)	29 (69)	23 (54)
Race or ethnicity, n (%)												
White	NR	NR	NR	19 (91)	19 (100)	18 (90)	19 (95)	10 (100)	18 (90)	39 (91)	38 (91)	41 (95)
Black or African American	NR	NR	NR	1 (5)	0 (0)	2 (10)	1 (5)	0 (0)	1 (5)	2 (5)	1 (2)	1 (2)
Asian	NR	NR	NR	1 (5)	0 (0)	0 (0)	0 (0)	0 (0)	0 (0)	2 (5)	3 (7)	0 (0)
Native Hawaiian or other Pacific Islander	NR	NR	NR	0 (0)	0 (0)	0 (0)	0 (0)	0 (0)	1 (5)	0 (0)	0 (0)	0 (0)
Other	NR	NR	NR	0 (0)	0 (0)	0 (0)	0 (0)	0 (0)	0 (0)	0 (0)	0 (0)	1 (2)
Hispanic or Latino	NR	NR	NR	10 (48)	12 (63)	11 (55)	7 (35)	5 (50)	8 (40)	15 (35)	17 (40)	20 (47)
Metabolic risk factors and parameters, mean (SD)												
Body weight, kg	102	99	95	99.6 (15.3)	108.2 (25.3)	103.6 (26.2)	103.1 (20.4)	119.1 (30.5)	97.9 (19.8)	107.6 (25.6)	103.9 (22.7)	102.8 (21.1)
Body mass index, kg/m2	NR	NR	NR	37.6 (4.8)	38.8 (9.3)	36.7 (6.8)	37.2 (5.5)	39.1 (8.2)	36.0 (5.6)	38.7 (7.7)	38.3 (6.9)	37.2 (6.6)
Type 2 diabetes, n (%)	50 (82)	46 (81)	49 (78)	14 (67)	7 (37)	10 (50)	10 (50)	5 (50)	10 (50)	28 (65·1)	32 (76·2)	30 (69·8)
Hemoglobin A1c, %	6.8	6.8	6.6	6.49 (1.0)	6.20 (1.0)	6.43 (1.2)	6.23 (1.2)	6.6 (1.4)	6.1 (1.0)	6.8 (1.1)	6.8 (1.0)	6.7 (1.2)
Liver Histology												
Patients with F1, n (%)	0 (0)	0 (0)	0 (0)	8 (38)	7 (37)	7 (35)	7 (35)	0 (0)	0 (0)	0 (0)	0 (0)	0 (0)
Patients with F2, n (%)	0 (0)	0 (0)	0 (0)	5 (24)	7 (37)	8 (40)	6 (30)	0 (0)	0 (0)	13 (30%)	15 (36%)	16 (37%)
Patients with F3, n (%)	0 (0)	0 (0)	0 (0)	8 (38)	5 (26)	5 (25)	7 (35)	0 (0)	0 (0)	30 (70)	27 (64)	27 (63)
Patients with F4, n (%)	61 (100)	57 (100)	63 (100)	0 (0)	0 (0)	0 (0)	0 (0)	10 (100)	20 (100)	0 (0)	0 (0)	0 (0)
MASLD activity score, mean (SD)	NR	NR	NR	5.1 (1.0)	5.6 (1.0)	5.1 (1.2)	5.6 (0.7)	3.3 (2.1)	4.1 (1.7)	5.4 (1.2)	5.1 (1.0)	5.6 (1.1)
Markers of fibrosis, mean (SD)												
Pro-C3, μg/L	132	142	147	16.1 (6.7)	19.2 (10.7)	16.2 (5.8)	17.2 (5.9)	22.6 (11.8)	25.6 (27.5)	16.5 (6.1)	15.3 (5.5)	18.4 (8.0)
ELF score	10.4	10.6	10.5	9.5 (1.0)	9.5 (0.6)	9.5 (0.9)	9.5 (0.8)	9.7 (0.8)	10.4 (1.2)	9.8 (0.7)	9.7 (0.8)	9.8 (0.8)
Liver stiffness, kPA	24.7	24.1	24.5	NR	NR	NR	NR	25.8 (13.2)	22.1 (10.8)	14.5 (6.2)	13.8 (5.2)	16.0 (7.1)
Outcome indicator (extractable data)	(1)			(1) (5) (6)				(1) (2) (3) (4) (5) (6)		(1) (2) (3) (4) (5)		

SD: standard deviation

NR:not reported

ProC3: N-terminal type-III, collagen pro-peptide

ELF, score: Enhanced liver fibrosis score

Median liver stiffness by vibration-controlled transient elastography (FibroScan), kPa

(1) The proportion of patients with improvement in liver fibrosis by 1 or more stages without worsening of MASH.

(2) Enhanced liver fibrosis [ELF] score

(3) N-terminal type-III, collagen pro-peptide [ProC3].

(4) Liver stiffness by FibroScan.

(5) Treatment-emergent adverse events (TEAEs).

(6) Drug-related TEAEs.

### 3.2 Risk of bias in studies

**FIGURE 2:**
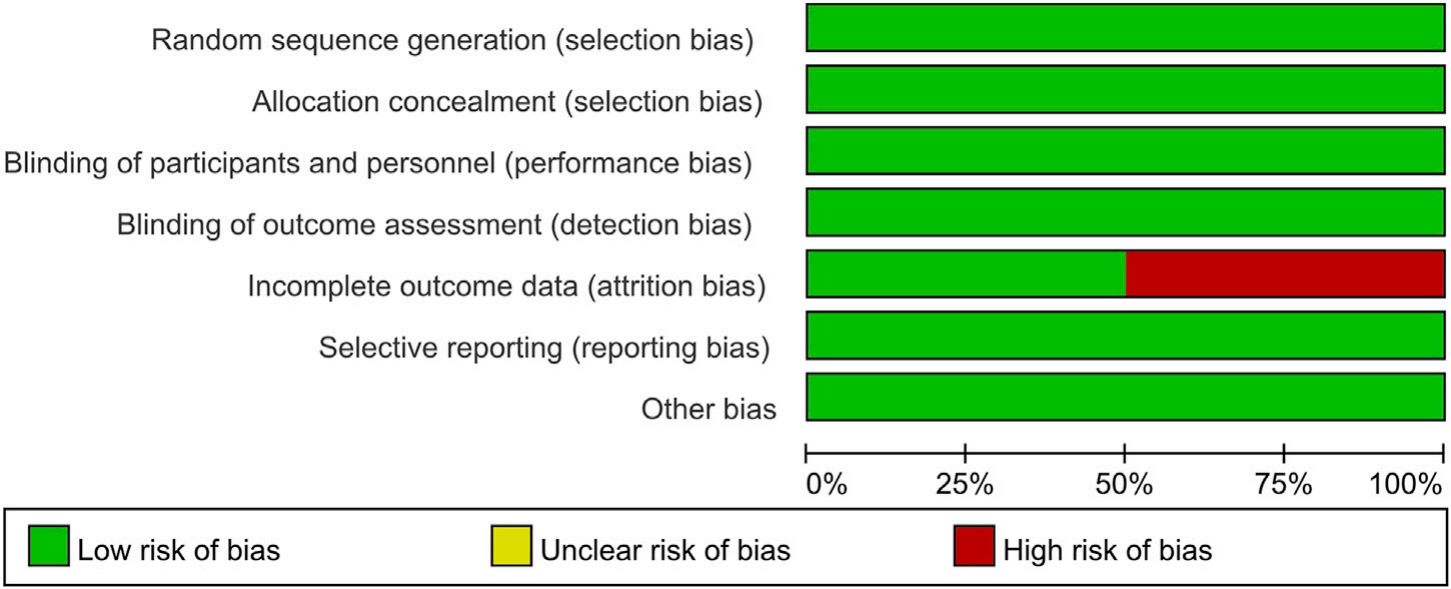
Risk of bias summary: review authors’ judgments about each risk of bias item for each included study. Seven domains (e.g., random sequence generation, allocation concealment, and blinding of participants) were categorized as low, unclear, or high risk, with percentage bars (0%–100%) reflecting the distribution of studies across bias categories.

**FIGURE 3:**
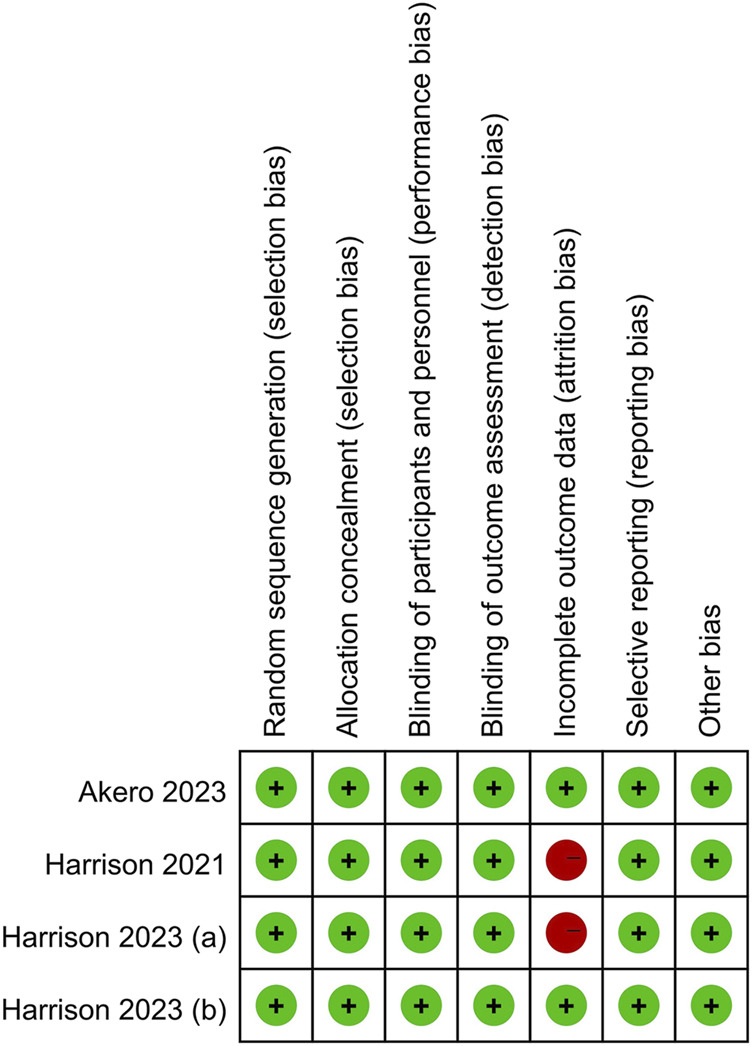
Risk of bias summary: review authors’ judgments about each risk of bias item for each included study. This figure summarizes the risk of bias assessment across seven domains for four studies. Harrison (2021) and Harrison (2023a) exhibited a high risk of bias in incomplete outcome data (attrition bias), with all other domains assessed as low risk.

The Cochrane Collaboration tool was used to assess the quality of the included studies and evaluate the risk of bias. Two studies had a high risk of attrition bias (Harrison et al., 2021; Harrison et al., 2023b). One study reported a 16% loss of biopsy data at the end of the trial due to restrictions caused by the COVID-19 pandemic (Harrison et al., 2021). The other study had attrition bias caused by an imbalance in missing data across groups (Harrison et al., 2023b). The remaining studies had a low risk of bias for all domains. Therefore, incomplete outcome data had a 50% high risk of bias and a 50% low risk of bias. The risk of selection bias, performance bias, detection bias, reporting bias, and other biases was low across all studies. Figures 2, 3 presented the results of the risk of bias assessment.

### 3.3 Results of syntheses

#### 3.3.1 Primary outcome

##### 3.3.1.1 The proportion of patients with improvement in liver fibrosis by 1 or more stages without worsening of MASH

**FIGURE 4:**
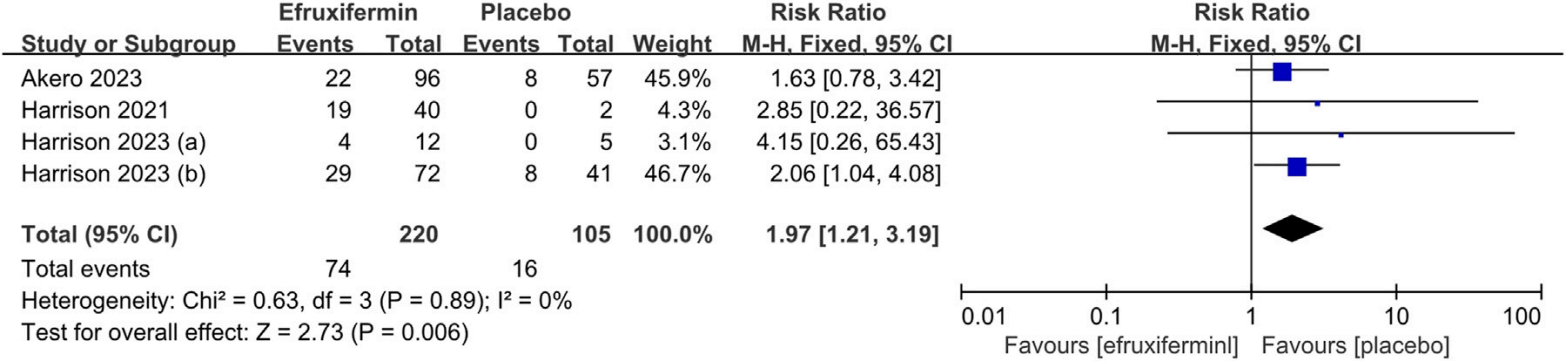
Results of the proportion of patients with improvement in liver fibrosis by 1 or more stages without worsening of MASH: efruxifermin vs. placebo. Pooled analysis demonstrated a significant risk ratio of 1.97 (95% CI 1.21–3.19; *P* = 0.006) favoring efruxifermin, with no heterogeneity observed (*I* ^*2*^ = 0%). CI, confidence interval; df, degrees of freedom.

A meta-analysis of 4 studies with a total of 325 patients was conducted on the proportion of patients who had improvement in fibrosis stage by ≥ 1 stage without worsening of MASH (Harrison et al., 2021; Harrison et al., 2023a; Harrison et al., 2023b). Two of the four studies had a high risk of attrition bias (Harrison et al., 2021; Harrison et al., 2023b). As shown in Figure 4, efruxifermin demonstrated superior efficacy over placebo in the proportion of patients with improvement in liver fibrosis by 1 or more stages without worsening of MASH (RR = 1.97, 95% CI 1.21 to 3.19, *P* = 0.006). The *I* ^*2*^ of 0% indicated no statistical heterogeneity, and thus the fixed-effects model was used.

#### 3.3.2 Secondary outcomes

##### 3.3.2.1 ELF score, ProC3, and liver stiffness by FibroScan

We performed meta-analyses on ELF score, ProC3, and Liver stiffness by FibroScan, respectively. Two RCTs were included in each analysis (Harrison et al., 2023a; Harrison et al., 2023b). One study had a high risk of attrition bias (Harrison et al., 2023b).

**FIGURE 5:**
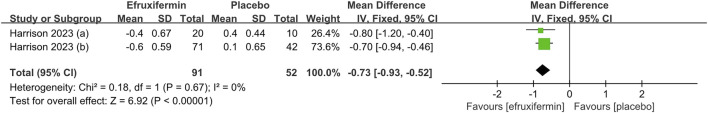
Results of ELF score: efruxifermin vs. placebo. Pooled analysis demonstrated a significant risk ratio of −0.73 (95% CI -0.93 to -0.52 P < 0.00001) favoring efruxifermin, with no heterogeneity observed (I^2^ = 0%). CI, confidence interval; df, degrees of freedom.

**FIGURE 6:**
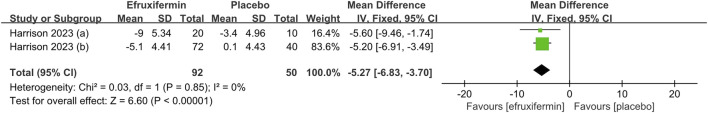
Results of Pro-C3: efruxifermin vs. placebo. Pooled analysis demonstrated a significant risk ratio of −5.27 (95% CI -6.83 to -3.70; P < 0.00001) favoring efruxifermin, with no heterogeneity observed (I^2^ = 0%). CI, confidence interval; df, degrees of freedom.

**FIGURE 7:**
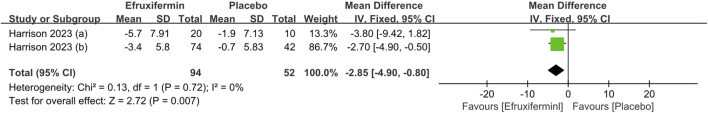
Results of Liver stiffness by FibroScan: efruxifermin vs. placebo. Pooled analysis demonstrated a significant risk ratio of −2.85 (95% CI -4.90 to -0.80; P = 0.007) favoring efruxifermin, with no heterogeneity observed (I^2^ = 0%). CI, confidence interval; df, degrees of freedom.

As shown in the figure, efruxifermin significantly lowered ELF score compared to placebo (MD -0.73, 95% CI: -0.93 to −0.52, *P* < 0.00001). For the reduction of ProC3, efruxifermin demonstrated superior efficacy over placebo (MD -5.27, 95% CI: -6.83 to −3.70, *P* < 0.00001). A similar advantage was observed in improving liver stiffness (MD = −2.85, 95% CI: -4.90 to −0.80, *P* = 0.007). No statistical heterogeneity was presented across the included studies for all analyses (*I* ^*2*^ = 0%). Details can be found in the Figures 5–7.

##### 3.3.2.2 TEAEs and drug-related TEAEs

**FIGURE 8:**
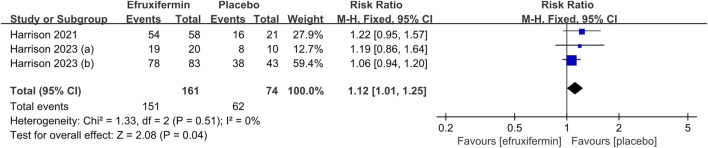
Results of Treatment-emergent adverse events (TEAEs): efruxifermin vs. placebo. Pooled analysis demonstrated a significant risk ratio of 1.12 (95% CI 1.01–1.25; P = 0.04) favoring efruxifermin, with no heterogeneity observed (I^2^ = 0%). CI, confidence interval; df, degrees of freedom.

**FIGURE 9:**
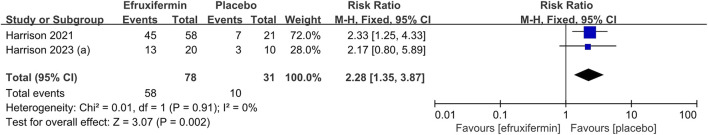
Results of Drug-related TEAEs: efruxifermin vs. placebo. Pooled analysis demonstrated a significant risk ratio of 2.28 (95% CI 1.35–3.87; P = 0.002) favoring efruxifermin, with no heterogeneity observed (I^2^ = 0%). CI, confidence interval; df, degrees of freedom.

For the analysis of TEAEs, we included three studies with a total of 235 patients (Harrison et al., 2021; Harrison et al., 2023a; Harrison et al., 2023b). Two studies had a high risk of attrition bias (Harrison et al., 2021; Harrison et al., 2023b). As depicted in Figure 8, the risk was significantly higher in the efruxifermin group compared to the control group (RR = 1.12, 95% CI 1.01 to 1.25, *P* = 0.04). A similar trend was observed for drug-related TEAEs (Figure 9), which also favored placebo (RR = 2.28, 95% CI 1.35 to 3.87, two RCTs, *P* = 0.002) (Harrison et al., 2021; Harrison et al., 2023b). There was no statistical heterogeneity detected across the included studies for all analyses (*I* ^*2*^ = 0%).

#### 3.3.3 Sensitivity analysis

To assess the robustness of the meta-analysis results, sensitivity analysis was conducted by changing the pooled effect model and iteratively excluding individual studies for the dichotomous outcomes (Supplementary Figures S1–S12). The statistical significance of all analyses remained unchanged after altering the pooled effect model, with no changes in heterogeneity (Supplementary Figures S1–S3). The beneficial effect on fibrosis improvement retained statistical significance regardless of which study was excluded. However, it was noteworthy that after excluding the Akero, 2023, the risk ratio increased from 1.97 to 2.25 (Supplementary Figure S4). For TEAEs, the difference became statistically insignificant (*P* = 0.17 > 0.05) after excluding Harrison 2021 (Harrison et al., 2021) (Supplementary Figure S8). A similar result was found for drug-related TEAEs (*P* = 0.13 > 0.05) (Supplementary Figure S11). This may be related to the grouping dose of Harrison et al. (2021).

#### 3.3.4 Publication bias

We only constructed a funnel plot for the primary outcome due to the limited inclusion of studies about secondary outcomes. From the plot, it is evident that the distribution of included studies was relatively symmetrical, suggesting a low likelihood of publication bias (Supplementary Figure S13).

#### 3.3.5 GRADE certainty of evidence

The GRADE evidence profile is presented in Supplementary Table S6. Except for the evidence on the proportion of patients with improvement in liver fibrosis by 1 or more stages without worsening of MASH’ being of moderate certainty, the certainty of the evidence for the remaining outcomes was low.

## 4 Discussion

In this systematic review and meta-analysis of four studies with a total of 325 patients, our analysis demonstrated that compared to placebo, efruxifermin consistently led to an improvement of at least one stage in liver fibrosis without worsening of MASH, meeting the FDA’s acceptable regulatory endpoint for approval of novel MASH therapies (Sanyal et al., 2011; Loomba et al., 2022). Efruxifermin also improved fibrosis-related non-invasive biomarkers. While it conferred an increased risk of primarily mild to moderate, transient gastrointestinal adverse events, this finding was not robust in sensitivity analysis.

Efruxifermin, a bivalent Fc-FGF21 analog, acts as a balanced agonist of FGFR1c, FGFR2c, and FGFR3c via β-Klotho co-receptor activation. By targeting FGFR1c in adipocytes, it suppresses lipolysis and enhances adiponectin secretion, reducing hepatic fatty acid influx, while FGFR2c/3c activation in hepatocytes inhibits *de novo* lipogenesis and lipid accumulation. This dual action enhances mitochondrial function, activates antioxidant pathways, and alleviates hepatocyte lipotoxicity, thereby reducing liver injury and inflammation. The drug improves systemic metabolic health by enhancing insulin sensitivity, normalizing lipid profiles (reducing atherogenic lipids and elevating HDL cholesterol), and directly suppressing hepatic stellate cell activation to mitigate fibrosis. Synergistically, efruxifermin complements GLP-1RAs by maintaining their metabolic benefits while accelerating hepatic fat normalization and fibrosis regression, supported by a sustained pharmacokinetic profile enabling once-weekly dosing (Harrison et al., 2021; Harrison et al., 2023a; Harrison et al., 2025).

Wang et al. (2024) highlighted that for the treatment of MASH, priority consideration may be given to thiazolidinediones (TZDs), vitamin E combined with pioglitazone, glucagon-like peptide-1 (GLP-1) receptor agonists, and FGF-21 analogs. Currently, FGF21 analogs under development for treating MASH mainly include pegozafermin, efruxifermin, and pegbelfermin. Results from a Phase 2b clinical trial revealed that pegbelfermin exhibited no statistically significant efficacy in treating MASH, prompting the discontinuation of its further development owing to its ineffectiveness (Loomba et al., 2024). Pegozafermin is a long-acting polyethylene glycolylated FGF21 analog. A randomized, double-blind, placebo-controlled Phase 1b/2a dose-escalation study demonstrated a significant reduction in hepatic fat with good tolerability and minimal adverse events in patients with MASH and stage F1–F3 fibrosis or those at high risk of MASLD and MASH (Loomba et al., 2023a). A Phase 2b clinical trial targeting biopsy-proven MASH patients with stage F2–F3 fibrosis showed improvement in fibrosis with pegozafermin treatment (Loomba et al., 2023b). For now, compared to pegozafermin, the study of efruxifermin included patients with MASH and F4 fibrosis, making its application more extensive (Harrison et al., 2023b).

A recent systematic review and network meta-analysis indicated that efruxifermin demonstrated significant efficacy for metabolic dysfunction-associated steatohepatitis (MASH) resolution without fibrosis worsening (risk ratio [RR]: 3.51, 95% credible interval [CrI]: 1.83–8.17; surface under the cumulative ranking curve [SUCRA]: 76.22), ranking fourth behind pegozafermin, survodutide, and tirzepatide, with moderate fibrosis improvement (RR: 1.85, 95% CrI: 1.30–2.75; SUCRA: 56.20), though its comparative efficacy against leading therapies and long-term durability require validation despite superior performance to placebo and approved agents (e.g., semaglutide, resmetirom) (Souza et al., 2025). Lin et al. (2024) published a meta-analysis investigating the efficacy and safety of FGF21 analogs in treating MASH and associated fibrosis. Their study included 9 RCTs comprising 1,054 patients with biopsy-proven MASH. Their findings suggested that FGF21 analogs appear promising in the treatment of MASH and MASH-related fibrosis, with generally good safety and tolerability profiles. In comparison to that study, our focus was primarily on efruxifermin, which possesses a longer half-life compared to most FGF21 analogs. Furthermore, our study placed greater emphasis on efruxifermin’s potential for improving liver fibrosis. Different from that study, the heterogeneity of the included studies in our study had less impact on the analysis results. Our findings suggested that efruxifermin may be a potential therapeutic agent for MASH-related fibrosis, with existing data indicating its favorable tolerability profile. These results provide robust evidence supporting the consideration of efruxifermin in larger-scale clinical trials for patients with MASH-related fibrosis.

Our meta-analysis has several notable strengths. To our knowledge, this is the first systematic review and meta-analysis specifically focusing on efruxifermin, and the included studies were multicenter, randomized, double-blind, placebo-controlled trials, yielding high generalizability. Moreover, our results demonstrated that efruxifermin significantly improved liver fibrosis in patients with MASH and stage F1–F4 fibrosis, indicating a broad applicability. Uniquely, our meta-analysis incorporated not only histological outcomes but also fibrosis-related non-invasive biomarkers, such as ELF score, ProC3, and liver stiffness, as well as tolerability data on adverse events. The findings from our study provide a strong rationale to further investigate efruxifermin in larger-scale clinical trials and inform its potential future clinical applications in treating MASH and MASH-related liver fibrosis.

Our study has several limitations. Firstly, due to the limited number of clinical trials on efruxifermin, with an ongoing phase 3 trial, we could only include a few studies, potentially compromising the reliability and accuracy of our meta-analysis. Future updates incorporating more trials are warranted. Secondly, the paucity of included studies precluded subgroup analyses. Thirdly, according to the results of sensitivity, the results of treatment-emergent adverse events and drug-related adverse events were not robust. Fourthly, as efruxifermin is an investigational drug, all included studies were sponsored by Akero Therapeutics, raising potential concerns of publication bias. The two studies have a high risk of attrition bias, which may bias the results to some extent. Fifthly, our analysis solely focused on the impact of efruxifermin on liver fibrosis, while its effects on other aspects, such as glycemic control, insulin sensitivity, lipid metabolism, and weight changes, were not evaluated.

In conclusion, efruxifermin may be a potential therapeutic for MASH-related fibrosis, with the available data indicating a seemingly favorable tolerability.

## 5 Impact and implications

To our knowledge, this is the first systematic review and meta-analysis specifically focusing on efruxifermin, and the included studies were multicenter, randomized, double-blind, placebo-controlled trials, yielding high generalizability. We found that efruxifermin may be a potential therapeutic for MASH-related fibrosis, with the available data indicating a seemingly favorable tolerability. Our study may provide some scientific support for drug selection in patients with NASH/MASH.

## Data Availability

The original contributions presented in the study are included in the article/Supplementary Material, further inquiries can be directed to the corresponding author.
