# Supplementary material for: A systematic review and meta-analysis of efruxifermin’s efficacy in improving liver fibrosis in patients with NASH/MASH
Source: Front Pharmacol. 2025 May 30;16:1594091. doi: 10.3389/fphar.2025.1594091 (PMC12163238; doi:10.3389/fphar.2025.1594091)
Supplement: Supplementary file 1 [file DataSheet1.pdf]

Supplementary Information

Table S1. PRISMA checklist.

Table S2. Database search strategy (PubMed).

Table S3. Database search strategy (Embase).

Table S4. Database search strategy (Cochrane).

Table S5. Read the full text but excluded studies.

Figures S1, S4-S7. Sensitivity analysis: the proportion of patients with improvement in liver fibrosis by 1 or more stages without worsening of NASH.

Figures S2, S8-S10. Sensitivity analysis: Treatment-emergent adverse events.

Figures S3, S11-S12. Sensitivity analysis: Drug-related TEAEs.

Figure S13. Funnel plot of the proportion of patients with improvement in liver fibrosis by 1 or more stages without worsening of NASH.

Table S6. The Grading of Recommendations Assessment, Development, and Evaluation (GRADE) evidence profile.

Table S1. PRISMA checklist.

Section and Topic	Item #	Checklist item	Location where item is reported
**TITLE**			
Title	1	Identify the report as a systematic review.	Title
**ABSTRACT**			
Abstract	2	See the PRISMA 2020 for Abstracts checklist.	Abstract
**INTRODUCTION**			
Rationale	3	Describe the rationale for the review in the context of existing knowledge.	Introduction
Objectives	4	Provide an explicit statement of the objective(s) or question(s) the review addresses.	Introduction
**METHODS**			
Eligibility criteria	5	Specify the inclusion and exclusion criteria for the review and how studies were grouped for the syntheses.	Eligibility criteria
Information sources	6	Specify all databases, registers, websites, organisations, reference lists and other sources searched or consulted to identify studies. Specify the date when each source was last searched or consulted.	Data collection and retrieval strategies
Search strategy	7	Present the full search strategies for all databases, registers and websites, including any filters and limits used.	Data collection and retrieval strategies, Table S2-S4
Selection process	8	Specify the methods used to decide whether a study met the inclusion criteria of the review, including how many reviewers screened each record and each report retrieved, whether they worked independently, and if applicable, details of automation tools used in the process.	Selection process and data extraction
Data collection process	9	Specify the methods used to collect data from reports, including how many reviewers collected data from each report, whether they worked independently, any processes for obtaining or confirming data from study investigators, and if applicable, details of automation tools used in the process.	Selection process and data extraction
Data items	10a	List and define all outcomes for which data were sought. Specify whether all results that were compatible with each outcome domain in each study were sought (e.g. for all measures, time points, analyses), and if not, the methods used to decide which results to collect.	Selection process and data extraction, Table 1
	10b	List and define all other variables for which data were sought (e.g. participant and intervention characteristics, funding sources). Describe any assumptions made about any missing or unclear information.	Table 1
Study risk of bias assessment	11	Specify the methods used to assess risk of bias in the included studies, including details of the tool(s) used, how many reviewers assessed each study and whether they worked independently, and if applicable, details of automation tools used in the process.	Risk of bias in individual studies
Effect measures	12	Specify for each outcome the effect measure(s) (e.g. risk ratio, mean difference) used in the synthesis or presentation of results.	Data synthesis and statistical analysis
Synthesis methods	13a	Describe the processes used to decide which studies were eligible for each synthesis (e.g. tabulating the study intervention characteristics and comparing against the planned groups for each synthesis (item #5)).	Table 1
	13b	Describe any methods required to prepare the data for presentation or synthesis, such as handling of missing summary statistics, or data conversions.	Data synthesis and statistical analysis
	13c	Describe any methods used to tabulate or visually display results of individual studies and syntheses.	Data synthesis and statistical analysis
	13d	Describe any methods used to synthesize results and provide a rationale for the choice(s). If meta-analysis was performed, describe the model(s), method(s) to identify the presence and extent of statistical heterogeneity, and software package(s) used.	Data synthesis and statistical analysis
	13e	Describe any methods used to explore possible causes of heterogeneity among study results (e.g. subgroup analysis, meta-regression).	Data synthesis and statistical analysis
	13f	Describe any sensitivity analyses conducted to assess robustness of the synthesized results.	Data synthesis and statistical analysis
Reporting bias assessment	14	Describe any methods used to assess risk of bias due to missing results in a synthesis (arising from reporting biases).	NA

Section and Topic	Item #	Checklist item	Location where item is reported
Certainty assessment	15	Describe any methods used to assess certainty (or confidence) in the body of evidence for an outcome.	Data synthesis and statistical analysis
**RESULTS**			
Study selection	16a	Describe the results of the search and selection process, from the number of records identified in the search to the number of studies included in the review, ideally using a flow diagram.	Figure 1
	16b	Cite studies that might appear to meet the inclusion criteria, but which were excluded, and explain why they were excluded.	Table S5
Study characteristics	17	Cite each included study and present its characteristics.	Table 1
Risk of bias in studies	18	Present assessments of risk of bias for each included study.	Risk of Bias in Studies, Figure 2-3
Results of individual studies	19	For all outcomes, present, for each study: (a) summary statistics for each group (where appropriate) and (b) an effect estimate and its precision (e.g. confidence/credible interval), ideally using structured tables or plots.	Results of syntheses
Results of syntheses	20a	For each synthesis, briefly summarise the characteristics and risk of bias among contributing studies.	Results of syntheses
	20b	Present results of all statistical syntheses conducted. If meta-analysis was done, present for each the summary estimate and its precision (e.g. confidence/credible interval) and measures of statistical heterogeneity. If comparing groups, describe the direction of the effect.	Results of syntheses
	20c	Present results of all investigations of possible causes of heterogeneity among study results.	Results of syntheses
	20d	Present results of all sensitivity analyses conducted to assess the robustness of the synthesized results.	Sensitivity analysis, Figure S1-S12
Reporting biases	21	Present assessments of risk of bias due to missing results (arising from reporting biases) for each synthesis assessed.	NA
Certainty of evidence	22	Present assessments of certainty (or confidence) in the body of evidence for each outcome assessed.	GRADE certainty of evidence, Table S6
**DISCUSSION**			
Discussion	23a	Provide a general interpretation of the results in the context of other evidence.	Discussion
	23b	Discuss any limitations of the evidence included in the review.	Discussion
	23c	Discuss any limitations of the review processes used.	Discussion
	23d	Discuss implications of the results for practice, policy, and future research.	Discussion
**OTHER INFORMATION**			
Registration and protocol	24a	Provide registration information for the review, including register name and registration number, or state that the review was not registered.	Registration
	24b	Indicate where the review protocol can be accessed, or state that a protocol was not prepared.	Material and methods
	24c	Describe and explain any amendments to information provided at registration or in the protocol.	Material and methods
Support	25	Describe sources of financial or non-financial support for the review, and the role of the funders or sponsors in the review.	Funding
Competing interests	26	Declare any competing interests of review authors.	Conflict of interest statement
Availability of data, code and other materials	27	Report which of the following are publicly available and where they can be found: template data collection forms; data extracted from included studies; data used for all analyses; analytic code; any other materials used in the review.	Data availability statement

Table S2. Database search strategy (PubMed).

#1	"Non-alcoholic Fatty Liver Disease"[Mesh]
#2	((((((((((((Non alcoholic Fatty Liver Disease[Title/Abstract]) OR (NAFLD[Title/Abstract])) OR (Nonalcoholic Fatty Liver

	Disease[Title/Abstract])) OR (Fatty Liver, Nonalcoholic[Title/Abstract])) OR (Fatty Livers, Nonalcoholic[Title/Abstract])) OR (Liver, Nonalcoholic Fatty[Title/Abstract])) OR (Livers, Nonalcoholic Fatty[Title/Abstract])) OR (Nonalcoholic Fatty Live[Title/Abstract])) OR (Nonalcoholic Fatty Livers[Title/Abstract])) OR (Nonalcoholic Steatohepatitis[Title/Abstract])) OR (Nonalcoholic Steatohepatitides[Title/Abstract])) OR (Steatohepatitides, Nonalcoholic[Title/Abstract])) OR (Steatohepatitis, Nonalcoholic[Title/Abstract])) OR (Nash[Title/Abstract])
#3	#1 OR #2
#4	((Efruxifermin[Title/Abstract]) OR (EFX[Title/Abstract])) OR (AKR-001[Title/Abstract])
#5	((randomized controlled trial[Title/Abstract]) OR (randomized[Title/Abstract])) OR (placebo[Title/Abstract])
#6	#3 AND #4 AND #5

Table S3. Database search strategy (Embase).

#1	'nonalcoholic fatty liver'/exp
#2	'non alcoholic fatty liver disease':ab,ti
#3	'non-alcoholic fatty liver disease':ab,ti
#4	'nafld':ab,ti
#5	'nonalcoholic fatty liver disease':ab,ti
#6	'fatty liver, nonalcoholic':ab,ti
#7	'fatty livers, nonalcoholic':ab,ti
#8	'liver, nonalcoholic fatty':ab,ti
#9	'livers, nonalcoholic fatty':ab,ti
#10	'nonalcoholic fatty liver':ab,ti
#11	'nonalcoholic fatty livers':ab,ti
#12	'nonalcoholic steatohepatitis':ab,ti
#13	'nonalcoholic steatohepatitides':ab,ti
#14	'steatohepatitides, nonalcoholic':ab,ti
#15	'steatohepatitis, nonalcoholic':ab,ti
#16	'nash':ab,ti
#17	#1 OR #2 OR #3 OR #4 OR #5 OR #6 OR #7 OR #8 OR #9 OR #10 OR #11 OR #12 OR #13 OR #14 OR #15 OR #16
#18	'efruxifermin'/exp
#19	'efx':ab,ti
#20	'akr-001':ab,ti
#21	#18 OR #19 OR #20
#22	'random':ti,ab
#23	'placebo':ti,ab
#24	'double-blind':ti,ab
#25	#22 OR #23 OR #24
#26	#17 AND #21 AND #25

Table S4. Database search strategy (Cochrane).

#1	MeSH descriptor: [Non-alcoholic Fatty Liver Disease] explode all trees
#2	(Non alcoholic Fatty Liver Disease):ti,ab,kw OR (NAFLD):ti,ab,kw OR (Nonalcoholic Fatty Liver Disease):ti,ab,kw OR (Fatty Liver, Nonalcoholic):ti,ab,kw OR (Fatty Livers, Nonalcoholic):ti,ab,kw
#3	(Liver, Nonalcoholic Fatty):ti,ab,kw OR (Livers, Nonalcoholic Fatty):ti,ab,kw OR (Nonalcoholic Fatty Liver):ti,ab,kw OR (Nonalcoholic Fatty Livers):ti,ab,kw OR (Nonalcoholic Steatohepatitis):ti,ab,kw (Word variations have been searched)
#4	(Nonalcoholic Steatohepatitides):ti,ab,kw OR (Steatohepatitides, Nonalcoholic):ti,ab,kw OR (Steatohepatitis,

	Nonalcoholic):ti,ab,kw OR (Nash):ti,ab,kw
#5	#1 or #2 or #3 or #4
#6	(Efruxifermin):ti,ab,kw OR (EFX):ti,ab,kw OR (AKR-001):ti,ab,kw
#7	(randomized controlled trial):pt OR (randomized):ti,ab,kw OR (placebo):ti,ab,kw
#8	#5 and #6 and #7

Table S5. Read the full text but excluded studies.

Title	Reason
Characterization of the patterns of resolution of histopathology after efruxifermin treatment of patients with NASH fibrosis (F2/3) for 24 weeks	Data from duplicate studies
EFRUXIFERMIN (EFX) IN NONALCOHOLIC STEATOHEPATITIS WITH FIBROSIS: RESULTS FROM A RANDOMIZED, DOUBLE-BLIND, PLACEBO-CONTROLLED, PHASE 2b TRIAL (HARMONY)	
Efruxifermin (EFX), a long-acting FC-FGF21 Fusion protein, administered for 16 weeks to patients with NASH substantially reduces liver fat and alt, and improves liver histology: analysis of a randomized, placebo-controlled, phase 2a study (balanced)	
A Study of AKR-001 in Subjects With Histologically Confirmed Nonalcoholic Steatohepatitis (NASH)	
A Study of Efruxifermin in Non-Cirrhotic Subjects With Histologically Confirmed Nonalcoholic Steatohepatitis (NASH)	
Non-invasive tests of liver injury, inflammation and fibrosis are improved by efruxifermin and correlate with histological improvements in F2-F3 NASH patients: secondary analysis of Ph2b HARMONY study	
Efruxifermin is associated with improved glucose metabolism in patients with NASH and type 2 diabetes	Studies without available data can be extracted
Increased adiponectin following efruxifermin treatment is associated with improvements in dyslipidemia, glucose metabolism, and liver health in a 16-week, randomized, placebocontrolled NASH trial	
A Study of Efruxifermin in Subjects With Compensated Cirrhosis Due to Nonalcoholic Steatohepatitis (NASH) (Symmetry)	
Efx treatment improved histopathology and non-invasive markers of liver injury and fibrogenesis to a similar extent in NASH patients with high-risk PNPLA3 genotypes, compared to those with lowest genetic risk: a post-hoc analysis of balanced study	

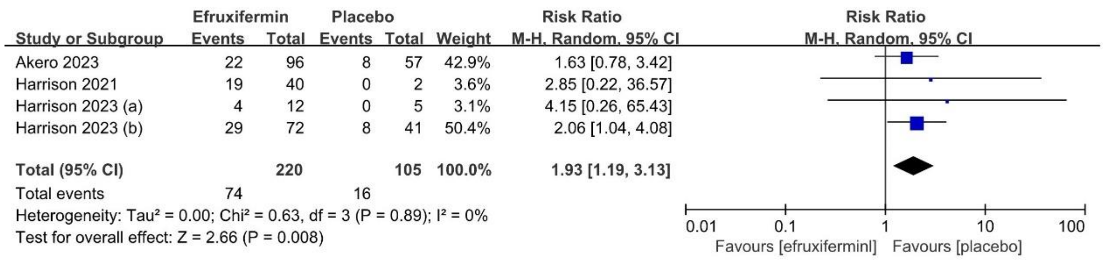

Figure S1. Sensitivity analysis: the proportion of patients with improvement in liver fibrosis by 1 or more stages without worsening of NASH. After switching from a fixed-effect model to a random-effect model, pooled analysis demonstrated a significant risk ratio of 1.19 (95% CI 1.19–3.13; *P* = 0.008) favoring efruxifermin, with no heterogeneity observed (I^2 = 0%). CI, confidence interval; df, degrees of freedom.

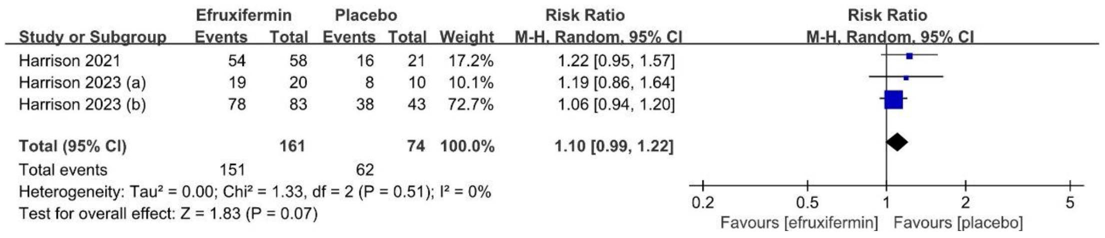

Figure S2. Sensitivity analysis: Treatment-emergent adverse events. After switching from a fixed-effect model to a random-effect model, the pooled analysis revealed a marginally non-significant risk reduction favoring efruxifermin (RR = 1.10, 95% CI 0.99–1.22; *P* = 0.07), with no evidence of between-study heterogeneity (I^2 = 0%). CI, confidence interval; df, degrees of freedom.

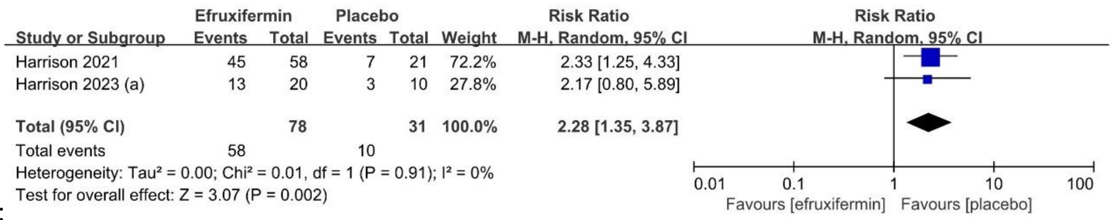

修改说明:

Figure S3. Sensitivity analysis: Drug-related TEAEs. After switching from a fixed-effect model to a random-effect model, pooled analysis demonstrated a significant risk ratio of 2.28 (95% CI 1.35–3.87; *P* = 0.002) favoring efruxifermin, with no heterogeneity observed (I^2 = 0%). CI, confidence interval; df, degrees of freedom.

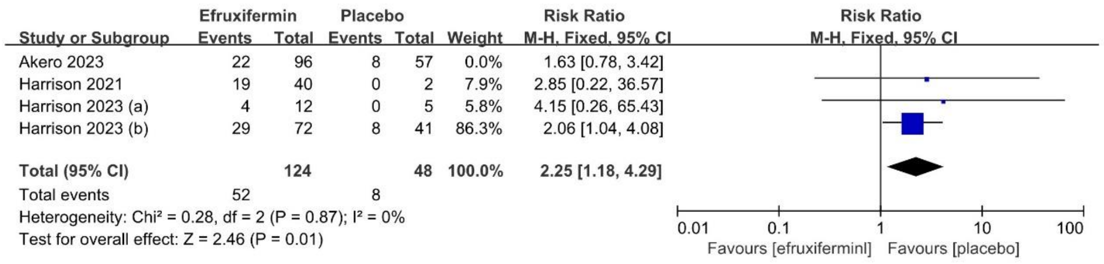

Figure S4. Sensitivity analysis: the proportion of patients with improvement in liver fibrosis by 1 or more stages without worsening of NASH. After excluding Akero 2023, the pooled analysis demonstrated a statistically significant risk ratio favoring efruxifermin (RR = 2.25, 95% CI 1.18–4.29; *P* = 0.01), with no evidence of heterogeneity (I^2 = 0%). CI, confidence interval; df, degrees of freedom.

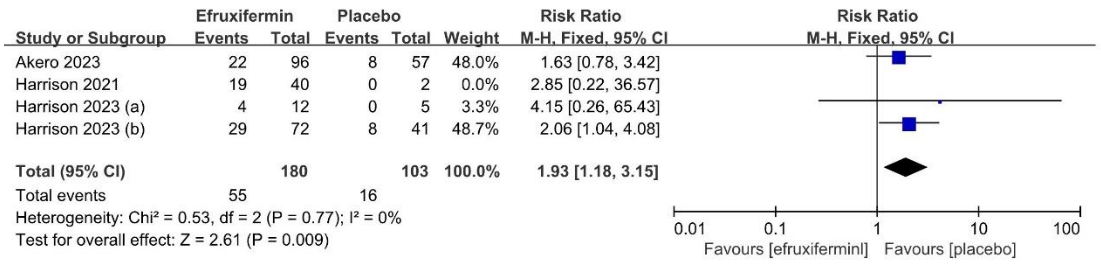

Figure S5. Sensitivity analysis: the proportion of patients with improvement in liver fibrosis by 1 or more stages without worsening of NASH. After excluding Harrison 2021, the pooled analysis demonstrated a statistically significant risk ratio favoring efruxifermin (RR = 1.93, 95% CI 1.18–3.15; *P* = 0.009), with no evidence of heterogeneity (I^2 = 0%). CI, confidence interval; df, degrees of freedom.

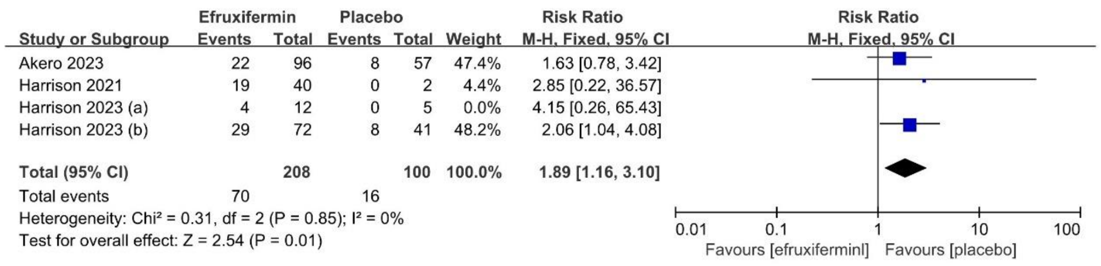

Figure S6. Sensitivity analysis: the proportion of patients with improvement in liver fibrosis by 1 or more stages without worsening of NASH. After

excluding Harrison 2023(a), the pooled analysis demonstrated a statistically significant risk ratio favoring efruxifermin (RR = 1.89, 95% CI 1.16–3.10; *P* = 0.01), with no evidence of heterogeneity (I^2 = 0%). CI, confidence interval; df, degrees of freedom.

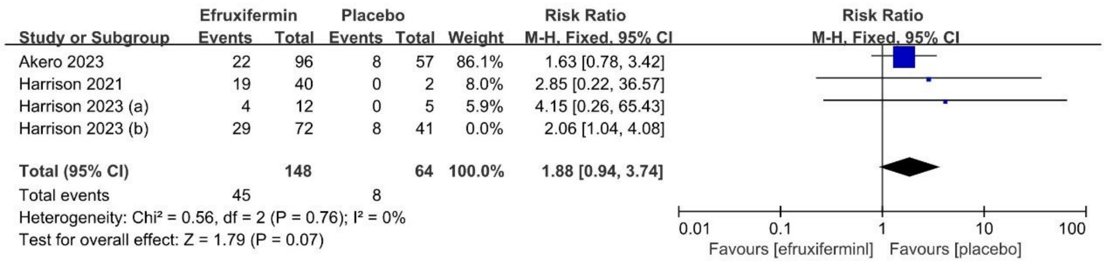

Figure S7. Sensitivity analysis: the proportion of patients with improvement in liver fibrosis by 1 or more stages without worsening of NASH. After excluding Harrison 2023(b), the pooled analysis revealed a marginally non-significant risk reduction favoring efruxifermin (RR = 1.88, 95% CI 0.94–3.74; *P* = 0.07), with no evidence of between-study heterogeneity (I^2 = 0%). CI, confidence interval; df, degrees of freedom.

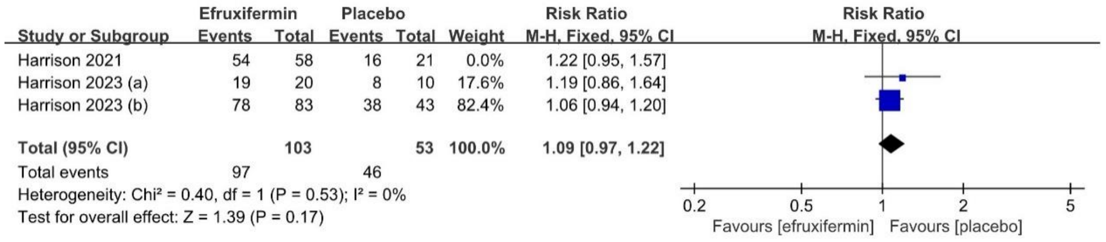

Figure S8. Sensitivity analysis: Treatment-emergent adverse events. After excluding Harrison 2021, the pooled analysis revealed a marginally non-significant risk reduction favoring efruxifermin (RR = 1.09, 95% CI 0.97–1.22; *P* = 0.17), with no evidence of between-study heterogeneity (I^2 = 0%). CI, confidence interval; df, degrees of freedom.

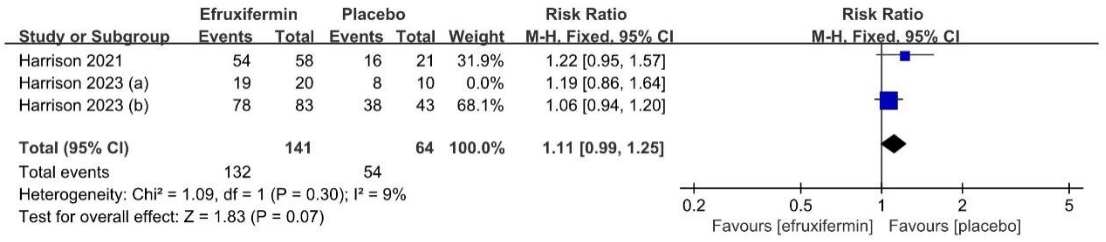

Figure S9. Sensitivity analysis: Treatment-emergent adverse events. After excluding Harrison 2023(a), the pooled analysis revealed a marginally non-significant risk reduction favoring efruxifermin (RR = 1.11, 95% CI 0.99–1.25; *P* = 0.07), with no evidence of between-study heterogeneity (I^2 = 0%). CI, confidence interval; df, degrees of freedom.

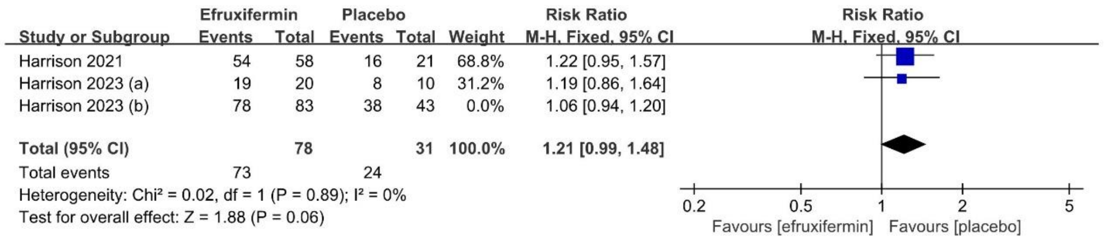

Figure S10. Sensitivity analysis: Treatment-emergent adverse events. After excluding Harrison 2023(b), the pooled analysis revealed a marginally non-significant risk reduction favoring efruxifermin (RR = 1.21, 95% CI 0.99–1.48; *P* = 0.06), with no evidence of between-study heterogeneity (I^2 = 0%). CI, confidence interval; df, degrees of freedom.

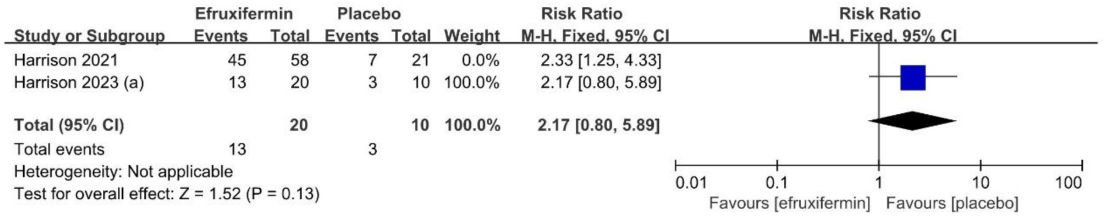

Figure S11. Sensitivity analysis: Drug-related TEAEs. After excluding Harrison 2021, the pooled analysis revealed a marginally non-significant risk reduction favoring efruxifermin (RR = 2.17, 95% CI 0.80–5.89; *P* = 0.13). CI, confidence interval; df, degrees of freedom.

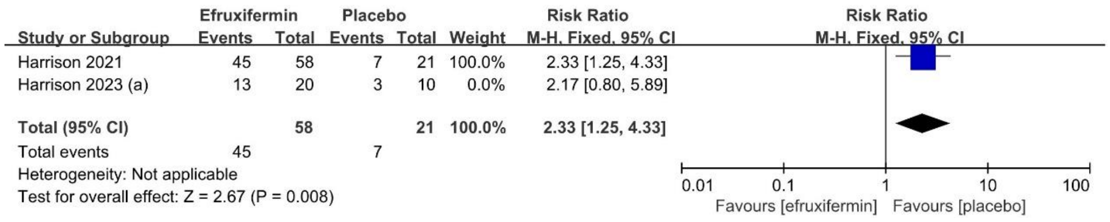

Figure S12. Sensitivity analysis: Drug-related TEAEs. After excluding Harrison 2023(a), the pooled analysis demonstrated a statistically significant risk ratio favoring efruxifermin (RR = 2.33, 95% CI 1.25–4.33; *P* = 0.008), with no evidence of heterogeneity (I^2 = 0%). CI, confidence interval; df, degrees of

freedom.

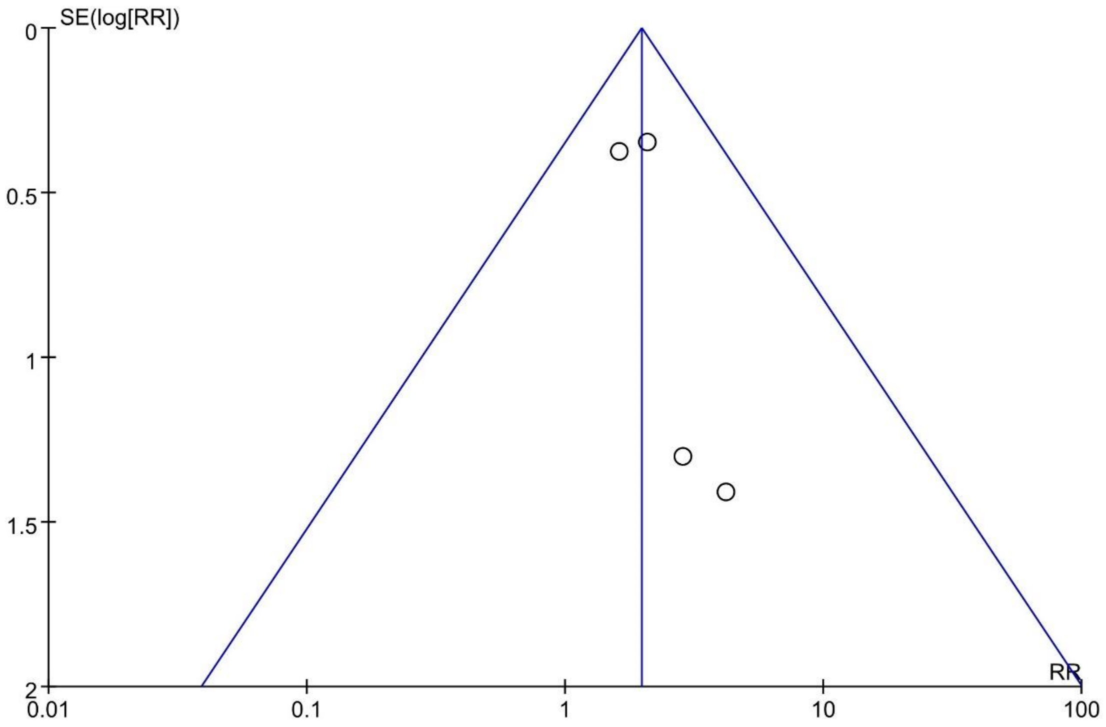

Figure S13. Funnel plot of the proportion of patients with improvement in liver fibrosis by 1 or more stages without worsening of NASH. It is evident that the distribution of included studies was relatively symmetrical, suggesting a low likelihood of publication bias.

Table S6. The Grading of Recommendations Assessment, Development, and Evaluation (GRADE) evidence profile. Except for the evidence on the proportion of patients with improvement in liver fibrosis by 1 or more stages without worsening of NASH' being of moderate certainty, the certainty of the evidence for the remaining outcomes was low.

Efruxifermin compared to Placebo for patients with non-alcoholic steatohepatitis (NASH)

Patient or population: patients with patients with non-alcoholic steatohepatitis (NASH)
Settings:
Intervention: Efruxifermin
Comparison: Placebo

Outcomes	Illustrative comparative risks* (95% CI)<br>Assumed risk<br>Placebo	 Corresponding risk<br>Efruxifermin	Relative effect (95% CI)	No of Participants (studies)	Quality of the evidence (GRADE)	Comments
**The proportion of patients with improvement in liver fibrosis by 1 or more stages without worsening of NASH**	**Study population**		**RR 1.97** (1.21 to 3.19)	325 (4 studies)	⊕⊕⊕⊖ **moderate**[1,2,3]	
	**152 per 1000**	**300 per 1000** (184 to 486)				
	**Moderate**					
	**70 per 1000**	**138 per 1000** (85 to 223)				
**Enhanced liver fibrosis [ELF] score**		The mean enhanced liver fibrosis [elf] score in the intervention groups was **0.73 lower** (0.93 to 0.52 lower)		143 (2 studies)	⊕⊕⊖⊖ **low**[4,5]	
**N-terminal type-III collagen pro-peptide [ProC3]**		The mean n-terminal type-iii collagen pro-peptide [proc3] in the intervention groups was **5.27 lower** (6.83 to 3.7 lower)		142 (2 studies)	⊕⊕⊖⊖ **low**[6,7]	
**Liver stiffness by FibroScan**		The mean liver stiffness by fibroscan in the intervention groups was **2.85 lower** (4.9 to 0.8 lower)		146 (2 studies)	⊕⊕⊖⊖ **low**[8,9]	
**Treatment-emergent adverse events (TEAEs)**	**Study population**		**RR 1.12** (1.01 to 1.25)	235 (3 studies)	⊕⊕⊖⊖ **low**[10,11]	
	**838 per 1000**	**938 per 1000** (846 to 1000)				
	**Moderate**					
	**800 per 1000**	**896 per 1000** (808 to 1000)				
**Drug-related TEAEs**	**Study population**		**RR 2.28** (1.35 to 3.87)	109 (2 studies)	⊕⊕⊖⊖ **low**[12,13]	
	**323 per 1000**	**735 per 1000** (435 to 1000)				
	**Moderate**					
	**317 per 1000**	**723 per 1000** (428 to 1000)				

*The basis for the **assumed risk** (e.g. the median control group risk across studies) is provided in footnotes. The **corresponding risk** (and its 95% confidence interval) is based on the assumed risk in the comparison group and the **relative effect** of the intervention (and its 95% CI).

CI: Confidence interval; **RR:** Risk ratio;

GRADE Working Group grades of evidence
High quality: Further research is very unlikely to change our confidence in the estimate of effect.
Moderate quality: Further research is likely to have an important impact on our confidence in the estimate of effect and may change the estimate.
Low quality: Further research is very likely to have an important impact on our confidence in the estimate of effect and is likely to change the estimate.
Very low quality: We are very uncertain about the estimate.

[1] Attrition bias was found in two studies
[2] The included studies had publication bias
[3] There were three studies with relative risk greater than 2 and no confounding factors
[4] One study had attrition bias
[5] There was publication bias in the included studies
[6] One study had attrition bias
[7] There was publication bias in the included studies
[8] One study had attrition bias
[9] There was publication bias in the included studies
[10] Attrition bias was found in two studies
[11] There was publication bias in the included studies
[12] One study had attrition bias
[13] There was publication bias in the included studies
